# Integrating Genetics and the Plasma Proteome to Predict the Risk of Type 2 Diabetes

**DOI:** 10.1007/s11892-020-01340-w

**Published:** 2020-10-08

**Authors:** Julia Carrasco Zanini, Maik Pietzner, Claudia Langenberg

**Affiliations:** MRC Epidemiology Unit, University of Cambridge, Cambridge, UK

**Keywords:** Plasma proteome, Genetics, Type 2 diabetes, Prediction, Causal risk factors

## Abstract

**Purpose of the Review:**

Proteins are the central layer of information transfer from genome to phenome and represent the largest class of drug targets. We review recent advances in high-throughput technologies that provide comprehensive, scalable profiling of the plasma proteome with the potential to improve prediction and mechanistic understanding of type 2 diabetes (T2D).

**Recent Findings:**

Technological and analytical advancements have enabled identification of novel protein biomarkers and signatures that help to address challenges of existing approaches to predict and screen for T2D. Genetic studies have so far revealed putative causal roles for only few of the proteins that have been linked to T2D, but ongoing large-scale genetic studies of the plasma proteome will help to address this and increase our understanding of aetiological pathways and mechanisms leading to diabetes.

**Summary:**

Studies of the human plasma proteome have started to elucidate its potential for T2D prediction and biomarker discovery. Future studies integrating genomic and proteomic data will provide opportunities to prioritise drug targets and identify pathways linking genetic predisposition to T2D development.

## Introduction

The global prevalence of diabetes, currently affecting 9.3% of the adult population, is predicted to increase up to 10.9% by 2045 [1]. This pandemic is largely attributable to an increase in the incidence of type 2 diabetes (T2D), the most common form of diabetes. A large proportion of affected adults do not have a clinical diagnosis [1], which can be delayed for several years after T2D onset [2, 3]. This leaves individuals with undiagnosed and untreated diabetes at high risk of developing severe and often irreversible microvascular and macrovascular complications [2, 4], and up to 30% of patients with T2D have been reported to present with evidence of retinopathy at the time of their diagnosis [5].

Criteria for screening and diagnosis of diabetes are focussed on glycaemic control, and guidelines recommend measurement of fasting glucose and HbA1c [6, 7]. The risk of developing future T2D can be relatively well predicted using simple, non-invasive measures such as age, sex, body mass index, and family history, and a range of algorithms have been tested and compared [8]. Over the last decade, genetic studies have greatly advanced our understanding of the polygenetic architecture of T2D, but with little evidence so far for improved prediction [9, 10] or genetically targeted strategies for prevention and treatment of T2D [11, 12].

The comprehensive assessment of the entirety of biomolecules across different layers of biological information, i.e. the genome, transcriptome, proteome, and metabolome, commonly referred to as the *–omics*, is now applied at scale in clinical and population-based settings to identify novel disease pathways. Proteins are the main effector molecules on cellular function, representing the largest class of pharmaceutical drug targets [13]. Over 150 FDA-approved biomarkers and diagnostic laboratory tests are based on plasma proteins [14], providing a source of clinically translatable discoveries. As the central layer of biological information transfer, the proteome can help to bridge the gap in our understanding of how genetic variation affects disease risk, including T2D and related metabolic disorders. Systematic identification of novel protein biomarkers and signatures also has the potential to improve targeted approaches for screening, diagnosis, and treatment and inform our understanding of disease heterogeneity and subtypes [15]. Comprehensive profiling of the circulating proteome has only recently been implemented in epidemiological studies due to challenges such as high cost, long measurement times, lack of scalable approaches, and the large dynamic range of the plasma proteome spanning almost ten orders of magnitude [16].

In this review, we describe the potential of plasma proteomics for T2D prediction, building on existing strategies and previous work focusing on the role of genetic risk. We summarise recent proteomic studies and their contribution to T2D prediction, biomarker discovery, and genetically informed target prioritisation. We searched PubMed and established preprint servers (BioRxiv/MedRxiv) for published studies that used high-throughput plasma proteomic profiling for T2D prediction, protein to T2D association studies, or integrated genetic data for causal assessment of proteins in Mendelian randomisation (MR) studies of T2D or related traits.

## Existing Strategies for T2D Prediction, Screening, and Diagnosis

Early preventative interventions have been shown to halt or delay T2D onset [17] and reduce long-term morbidity and mortality in individuals with impaired glucose tolerance [18], demonstrating the benefit of strategies targeting high risk individuals. Non-invasive scores, such as the Cambridge Diabetes Risk Score [19], have been proposed as practical and cost-effective tools to identify high-risk individuals [20]. These rely on an individual’s age, sex, ethnic origin, family history, medication use anthropometric and behavioural factors, which achieve good discrimination (C-statistics ranging from 0.76 to 0.81), and no strong evidence for superior performance of one score over another [21, 22]. Invasively obtained clinical predictors, such as glucose, glycated haemoglobin (HbA1c), blood lipids, or uric acid, improve the C-statistics (up to 0.90) [8] but with associated cost and organisational burden of obtaining blood samples.

Current guidelines for the diagnosis of T2D suggest the use of HbA1c or fasting plasma glucose [23], with thresholds originally determined on the basis of the risk of diabetic retinopathy [24–26]. An oral glucose tolerance test (OGTT) measures plasma glucose 2 hours after a standard glucose challenge (2hPG) and elevated levels of 2hPG are a major risk factor for diabetic retinopathy [25], but are not widely measured in the clinic due to the inconvenience for patients and healthcare professionals of the challenge test. This means that individuals with impaired glucose tolerance (IGT) and specifically isolated IGT and isolated post-challenge hyperglycaemia (IPH, i.e. normal fasting glucose but elevated 2hPG) are missed by current diagnostic strategies and are at greater risk of underdiagnosis and severe complications caused by their untreated chronic hyperglycaemia [27, 28]. IPH has been estimated to account for up to 60% of undiagnosed diabetes [29, 30] and is poorly predicted by existing scores and algorithms based on traditional clinical risk factors.

The tests recommended by current guidelines predominantly capture and focus on aspects of glucose metabolism. This is only one, relatively late alteration of the heterogeneous metabolic changes that are associated with T2D, which also involve hepatic lipid metabolism, adipose tissue accumulation, distribution and dysfunction, and inflammatory responses [31]. The current criteria for the definition of diabetes therefore do not fully reflect the existing aetiological heterogeneity and subtypes of T2D with consequences for prediction, screening, diagnosis, and ultimately prognosis. Whether more comprehensive metabolic or proteomic profiling could help to identify and target some of the larger existing subtypes, such as isolated IGT, and present a cost-effective strategy, remains uncertain.

### Polygenic Susceptibility and its Contribution to T2D Prediction

Over the past decade, genome-wide association studies (GWAS) have revealed the polygenic basis of T2D based on the identification of rare-to-common DNA sequence variations in the human genome [11, 32]. Over 400 distinct T2D signals have been published to date [33, 34], with common variants of small effects jointly explaining around 18% of the (chip-based) heritability, almost half of the heritability estimated from twin and family studies [33]. Genetic studies of T2D intermediate phenotypes (such as plasma glucose, insulin, and HbA1c) have identified regions involved in glycaemic regulation in non-diabetic individuals [35–37], many but not all of which also increase the risk of T2D [33, 35]. This complementary approach has greatly advanced our understanding of pathways leading to T2D development, previously reviewed in more detail [11].

Polygenic scores that combine risk alleles across T2D variants have been used as a measure for assessing genetic susceptibility or predisposition to T2D and identifying individuals at high risk [38]. However, there is little evidence for a clinically meaningful improvement of T2D prediction over and above simple and cheap (non-invasive) prediction models [11, 38]. There is substantial interest in the identification of distinct diabetes ‘subtypes’, and studies have proposed both genetic [39] and clinical risk factors [40–42] for their identification and classification. Partitioned polygenic scores have been developed by assigning subsets of variants to specific pathophysiological categories, such as insulin resistance, adiposity, or insulin secretion [39], and proposed as a tool to enable classification of patients to specific disease subtypes. However, this approach does not align well with what has been proposed based on biomarkers of newly diagnosed T2D patients [41], and there is currently no consensus on definitive T2D subcategories. It remains to be established whether these genetic approaches will provide new and actionable clinical insights for the identification and management of patients with diverse aetiologies over and above established risk factors [42]. While human genetics clearly offers translational opportunities, the integration of other -*omics* layers of information promises to improve T2D risk prediction beyond what can currently be achieved, by identifying individuals and population subgroups at sufficiently high risk of developing future diabetes that are not well captured with conventional approaches and poorly characterised by common variants with small effects.

### The Contribution of Plasma Proteomics to T2D Prediction

Early efforts to identify novel T2D biomarkers used prior biological knowledge for targeted assessment of mostly blood and immunoassay-based candidate biomarkers in observational association studies of prevalent or incident T2D. A systematic review described 167 protein, metabolite, or clinical biomarkers [43] for T2D, but established that their predictive value had only been evaluated for a small subset, with evidence for predictive utility for uric acid as the only non-glycaemic marker. This together with the lack of external validation and assay standardisation for new biomarkers has limited the translational value of previous studies, and there is currently no robust evidence of their added clinical value. Future studies that maximise biomarker coverage and thereby enable hypothesis-free approaches and analytical methods in prospective and sufficiently powered studies are required to systematically assess the potential of blood-based biomarkers to refine T2D prediction and classification beyond what is currently known.

The plasma proteome provides a snapshot of human physiology by integrating contributions from various tissues [44] and effectors such as genetic predisposition, medication, lifestyle, and undiagnosed or prevalent disease status [45]. It therefore provides opportunities for discoveries with high translational potential through (1) improved aetiological understanding, (2) development of risk assessment and stratification strategies based on the present state of the organism (as opposed to germline genetic variation), and (3) identification of novel pathways for intervention and prioritisation of drug targets, as most drugs act on human proteins [13]. High-throughput *proteomic* technologies with broad coverage of the proteome have only relatively recently become more widely available, compared with untargeted technologies assessing the *metabolome*, for example [46, 47]. Earlier challenges include plasma concentrations spanning several orders of magnitude, with high abundance proteins (including albumin and immunoglobulins) making up ~ 99% of total plasma proteome mass [48], technical difficulties in detecting low abundance proteins, such as cytokines and hormones, and achieving a balance between increasing the number of proteins measured while retaining target specificity.

**Fig. 1:**
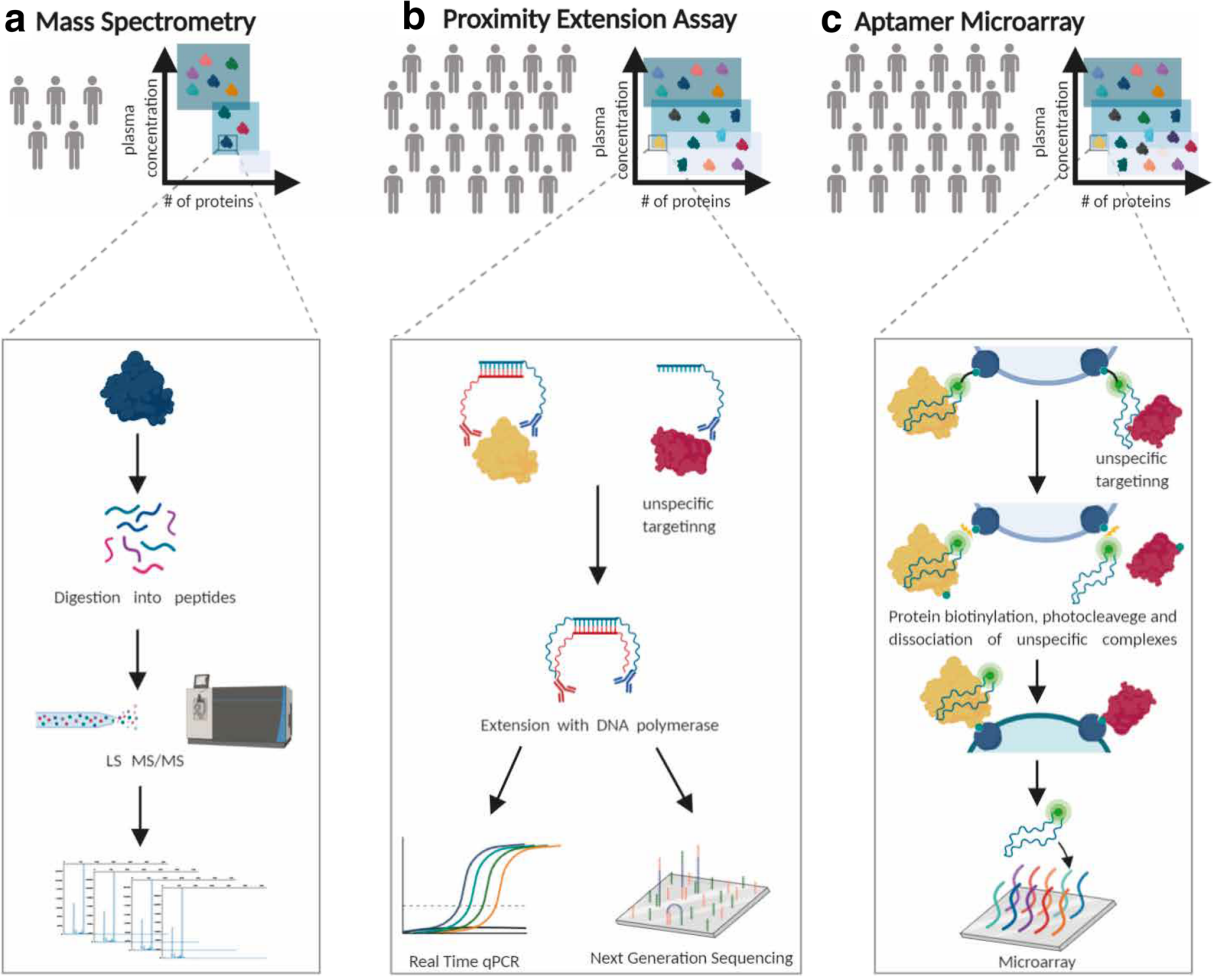
High-throughput proteomic technologies. **a** Mass spectrometry (MS)-based techniques rely on peptide sequences and are highly specific. However, they have limited throughput and mainly cover medium to high abundance plasma proteins. **b** Proximity extension assay (PEA) has increased throughput and coverage. Dual antibody targeting coupled to complementary oligonucleotides increases specificity and multiplexing capabilities of conventional immunoassays up to 92-plex or 384-plex panels quantified by qPCR or next-generation sequencing (NGS), respectively. Olink provides assays for over 1500 human proteins. **c** Aptamer microarrays have equally high throughput and coverage, capturing the full abundance spectrum in plasma but has increased multiplexing capabilities compared to PEA. Aptamers contain a fluorescent tag (green), cleavable link (black), and biotin (turquoise). Aptamer-bound proteins are biotinylated, and aptamer-protein complexes are released by photocleavage. Unspecific aptamer-protein binding is addressed by incubation with an anionic competitor that preferentially causes dissociation of non-specific complexes. Aptamer-protein complexes are captured onto streptavidin-coated beads, and aptamer still bound is released and quantified by hybridisation to microarrays. The SomaScan assay provides measures for over 5000 proteins (some being targeted by more than one aptamer). Figure created with BioRender.com

*Proteomic* technologies can be broadly divided into mass spectrometry (MS) and affinity-based assays. MS can be classified into targeted or untargeted (providing larger proteome coverage) and is considered the gold standard for multiple protein detection and measurement as it depends on peptide masses and sequences making it highly specific [49]. It further enables detection of post-translational modifications that regulate proteins’ biological activity, of which phosphorylation has been the most widely studied. High-throughput application of MS to human plasma faces several challenges. It is a labour-intensive multi-step technique, making it harder to reproducibly scale up for large epidemiological studies. A major disadvantage of untargeted approaches is the selected coverage of only moderate to highly abundant proteins, such as coagulation factors, immunoglobulins, or carrier proteins (Fig. 1a). Plasma fractionation [50], targeted methods such as isobaric tags for relative and absolute quantification (iTRAQ) [51], and coupling with an affinity-based step that enables capture and enrichment of specific targets [52] have been shown to increase coverage of low abundance proteins. The successful development of a novel MS methodology that couples a modified sample preparation pipeline with short-gradient high-flow liquid chromatography has been shown to increase throughput and reproducibility and now has the potential to enable high-precision proteomic profiling of hundreds of samples per day at low cost and is predicted to enhance the phenotypic characterisation of large-scale population-based studies soon [53].

The two major high-throughput affinity-based technologies, proximity extension assay (PEA) [54] as implemented by Olink® and multiplex aptamer microarrays [55] as implemented by SomaLogic®, capture a much wider dynamic range specifically improving detection at the lower end of the abundance spectrum including signalling and effector proteins. PEA targets each protein by several polyclonal antibody pairs coupled to complementary oligonucleotides (Fig. 1b), overcoming previous multiplexing limitations of commonly used antibody-based techniques (such as ELISA) due to reagent cross-reactivity and consequent loss of specificity. Aptamer-based targeting (Fig. 1c) uses short single-stranded oligonucleotides, which fold into a spatial configuration that specifically binds to protein targets, and has further expanded multiplexing capabilities, now covering ~ 5000 proteins as implemented by the latest version of the SomaScan assay. Compared with MS, affinity-based assays are highly scalable and reproducible, with low intra-assay coefficients of variation. However, these methods cannot measure post-translational modifications or proteins for which there is no corresponding affinity reagent. In addition, genetic variants that cause a change on the tri-dimensional structure of the target protein’s region (i.e. protein altering variants) recognised by the antibody or aptamer, resulting in decreased (or increased) affinity, can lead to measurement biases in carriers of this genotype, known as epitope effects.

Early studies using high-throughput proteomic technologies identified only a few plasma proteins associated with incident T2D over follow-up times of up to 9.5 years (Table 1) [56–58]. Very recently, Gudmundosdottir V. et al. reported 99 plasma proteins associated with incident T2D in 2940 participants of whom 112 were incident cases over a 5-year follow-up time [59•]. Only one of these studies showed a modest improvement in discrimination by adding 3 proteins (MASP, ApoE, and CRP) to the standard clinical model (AUC increased from 0.75 to 0.77). Up to 142 plasma proteins have been associated with prevalent T2D [59•, 60, 61] in cross-sectional studies, which aim to identify differences between T2D patients and well-matched control samples or associations with glycaemic parameters, providing a cost-effective and simple prioritisation strategy. However, these studies suffer from reverse confounding, i.e. changes in the plasma proteome due to the widespread effect of insulin resistance on metabolism, and lack of rigorous assessment of predictive utility. Similarly, between four and 60 plasma proteins have been associated with measures of insulin resistance (IR) [56, 62–64] and pre-diabetes (IGT and/or impaired fasting glucose) [65, 66] (Table 1), implicating extracellular matrix components, inflammatory processes, the complement system, lipoprotein metabolism and the epithelial-mesenchymal transition pathway. Larger sample sizes and replication in independent prospective studies are needed to systematically assess specificity, i.e. the unique role of those proteins in T2D, and sensitivity, i.e. performance of such protein markers in diverse populations, of those markers.

**Table 1:** High-throughput proteomic association studies with intermediate phenotypes, prevalent, and incident T2D

Trait	Sample size (number of cases)	Proteomic technology (number of target proteins)	Number of target proteins associated with the outcome*	Reference
Prospective studies				
Incident T2D (9.5 years mean follow-up time)	1367 (111 incident T2D cases)	Olink (92)	2	[56]
Incident T2D (follow-up after 6.5 years)	892 (123 incident T2D cases)	MS (14)	2 (MASP and adiponectin)	[57]
Incident T2D (follow-up after 8 years)	1026 (146 incident T2D cases)	Olink (92)	7	[58]
Incident T2D (follow-up after 5 years)	2940 (112 incident T2D cases). Validation in 356 (179 incident T2D cases)	SOMAscan (4137)	99, none remained significant when adjusting for BMI	[59•]
IGT (follow-up after 3 years)	72 (36 incident IGT cases)	SOMAscan (1025)	60 in univariate analysis, 30 in multivariable analysis	[65]
HOMA IR (follow-up after 1 year)	42	MS (437 in average per individual)	40	[62]
Cross-sectional studies				
HOMA IR	1367	Olink (92)	7	[56]
Prevalent T2D	2467 (211 prevalent T2D cases)	Olink (249)	29	[60]
Prevalent T2D	528 (12% with prevalent T2D)	SOMAscan (~ 5000)	21	[61]
Prevalent T2D	4784 (654 prevalent T2D cases)	SOMAscan (4137)	142	[59•]
Pre-diabetes	439	MS (23)	4 (MASP, THBS1, GPLD1, and ApoA-IV)	[66]
IGT	80 (40 prevalent IGT cases)	SOMAscan (1025)	41 in univariate analysis, 18 in multivariate analysis	[65]
Disposition index	100	SOMAscan (1129)	17	[63]
HOMA IR	100	SOMAscan (1129)	22	[63]
IR	17 (8 IR individuals)	SOMAscan (1499)	44	[64]

*depending on the threshold used in the original study

### Analytical Advancements in Proteomics for T2D Prediction

The multifactorial aetiology of complex diseases such as T2D resulting from a mixture of genetic and environmental drivers of altered physiological mechanisms suggests that assessment of combinations or *signatures* of many biomarkers covering a large range of metabolic pathways may provide a more comprehensive picture of an individual’s present health status and/or future risk and allow identification of population subgroups who share similar aetiologies. Huth et al. [67] used a targeted proteomic approach, grouping 47 biomarkers into 19 pathways, to illustrate the contribution of different pathways to the percentage of explained variance in incident T2D, showing the insulin-like growth factor (IGF)/IGF-binding proteins (IGFBP) system and adipose-derived hormone pathways, as the largest contributors to T2D risk. However, one of the challenges for identifying signatures is the high correlation among the variables, which can lead to some degree of redundancy and biased risk estimates [68]. To address this, some studies have started to implement more sophisticated analytical methods such as machine learning-based feature selection [69••] or dimensionality reduction strategies [70]. In a recent proof-of-principle study, the authors demonstrated the predictive utility of coupling large-scale proteomic profiling with machine learning, to simultaneously provide informative signatures of 11 health status indicators [69••]. To predict conversion from pre-diabetes to T2D within 10 years, they identified a 365-protein signature that improved sensitivity over standard fasting and 2-h post-load glucose levels. Machine learning algorithms come with their own set of challenges, of which model overfitting is a major one when applied to these high-dimensional settings. The three-strep approach used by Williams et al. [69••], in which the data were divided into a training (to identify top informative features and/or models typically between 50 and 70% of the data), an optimisation (to tune top models’ parameters, between 15 and 25%), and a validation set (between 15 and 25%), has proven successful in reducing overfitting. Stability selection procedures during the first training step can further improve robustness of selected features [71]. For clinical purposes, however, a balance must be achieved between the number of biomarkers (as large signatures are unlikely to be measured in routine clinical settings) while retaining substantial improvements in model accuracy and specificity.

As the first candidate biomarkers are emerging from the high-throughput proteomic platforms, several questions must be addressed before translation into the clinic. Proteins encoded by the same gene can have several isoforms with distinct biological effects and target tissues. Similarly, post-translational modifications and processing can change protein expression levels or its biological activity. As affinity-based proteomic platforms are so far unable to detect and distinguish these differences among the plasma proteome, follow-up on the specific nature of biological effects is required for candidates identified by these technologies. Additionally, cross-reactivity and specificity remain an issue. Efforts to validate candidate proteins through systematic comparison between different platforms are on their way and will be crucial. Alternatively, integration with genetic information can provide a readily available tool to address some of these questions such as target specificity and potential epitope effects.

### The Future of Prediction Strategies

Technological developments driving assessment of other *omics*, such as epigenomics [72], transcriptomics [73], and metabolomics [74, 75], provide an unprecedented expansion in the molecular information that can be systematically studied for biomarker discovery at scale. Integration of different layers of information ‘flow’ promises to provide a more comprehensive picture of the biology of complex diseases [76]. Recent studies on a few individuals at high risk of T2D with repeated multi-*omics* measures [77••, 78] provide proof of principle of the utility of multi-*omics* to improve predictive modelling for insulin resistance and to identify individual disease trajectories that integrate molecular events across these layers of information. Outlier biomarker analysis at healthy baseline visits enabled identification of early molecular signatures of disease, which promises to improve characterisation of aetiological subtypes. However, the strong interindividual variability in several *omics* measures means that large sample sizes are required to reliably identify these molecular signatures.

### Beyond Prediction: Integration of Genomics and Proteomics to Identify Novel Causal Pathways

Biomarkers for which evidence for predictive utility exists do not necessarily reflect causal mechanisms since model performance strongly depends on effect sizes regardless of whether a biomarker is confounded or the consequence of the disease or its risk factors. However, elucidating underlying causal pathways can contribute to identification of early disease trajectories that could have clinical implications for classification, risk assessment, and management. Genetic approaches that attempt to assess causality of observational ‘statistical’ protein-disease associations [79] are built on the principle of randomisation used in controlled trials (RCTs), which aims to minimise systematic differences between the intervention and control group [80] that can lead to spurious or confounded associations. Analogous to the design of an RCT, ‘Mendelian Randomisation’ uses the ‘random allocation’ of genetic variants to minimise confounding and draw inference about the causal effect of a protein on a disease outcome. Only a few studies have applied this method to protein biomarkers in the context of targeted or candidate biomarkers and T2D [43], and have not provided strong evidence of causality for most proteins investigated [81], even in cases with consistent and statistically significant observed associations. Such is the case for C-reactive protein, which despite the strong positive association with T2D has been deemed likely non-causal. This further highlights the importance of integrating genetic information to prioritise protein targets for the purpose of identifying novel causal pathways and developing effective interventions.

Proteins are under tight genetic control as regulatory or structural variants can alter their expression level, biological activity, interaction properties, and multiple processes involved from synthesis to secretion. Integration of genomic variation with the plasma proteome can therefore inform biological as well as technical aspects, such as affinity-based reagents’ target specificity and potential binding artefacts [16]. Protein GWAS (pGWAS) have identified variants that modulate protein abundance in plasma [82•, 83, 84], termed protein quantitative trait loci (pQTLs), which can be defined as in *cis*, those located in or around the protein coding gene, or in *trans*, those located elsewhere in the genome. A list of pGWAS studies was originally published by Suhre et al. [74] and updated in their online web resource. Two of the most comprehensive studies used the SomaLogic platform and have identified 5553 exome array variants affecting 1931 proteins in 5457 individuals [83] and 1927 genome-wide variants affecting 1478 proteins in 3301 individuals [82•]. We recently expanded the genetic discovery for a subset of 179 proteins from the SomaLogic platform, including 45 proteins with no previously described pQTLs, in 10,708 individuals [85]. Larger efforts in terms of sample size, such as those by the SCALLOP consortium (www.scallop-consortium.com), are underway to expand pQTL discovery. Their first results in over 30,000 individuals identified 467 pQTLs for 85 proteins from the Olink CVD-I panel [84]. Of note, these efforts will enable systematic evaluation of consistency between proteomic platforms and provide orthogonal validation of correct protein targeting, supported by pQTLs located in or around the protein coding gene, and help identify epitope effects. Open access platforms will further facilitate interrogation and validation of results from pQTL discovery efforts, such as our recently published interactive webserver (https://omicscience.org/apps/covidpgwas/) for a subset of host proteins interacting with SARS-CoV-2 [85].

To use pQTLs as instrumental variables to assess the causal association between proteins and diseases, specific conditions must be met, in addition to the key assumptions underlying MR studies more generally [86]. *Cis*-pQTLs are characterised by large effects sizes on protein expression, compared with other more distal traits, and are less likely to violate the “no horizontal pleiotropy” assumption (i.e. effects on the outcome through other paths than the protein in question) than *trans*-pQTLs. The use of *cis*-pQTLs ensures that genetic variants are clearly and specifically linked to the protein of interest and not to other, possibly unwanted, phenotypes.

Systematic causal assessment for protein candidates on T2D had little success at first [56, 60, 87], and only recently, several proteins are suggested to be causally related to T2D (Table 2). WFIKKN2 is the only protein identified by more than one study with consistent effect directions. However, only 14 of these causal candidates are covered by both SomaScan and Olink platforms, which may account in part for the limited overlap in findings between studies. Furthermore, consistency between findings poses a challenge, due to different approaches for variant selection as instrumental variables. There are several outstanding challenges in protein MR studies which must be addressed to strengthen current evidence and expand the list of proteins causally involved in T2D development. First, owing to limited sample sizes, only a few pQTLs per protein have been identified so far (Table 2), compared with other exposures being investigated through MR, e.g. up to hundreds of variants are used to emulate the effect of obesity. However, pQTLs explain a much larger proportion of the variance in protein levels (more than 60% for IL-6sRa [85]) compared with the variance explained for complex traits. Larger studies and in different ethnic backgrounds will be required to expand pQTL discoveries. Second, there is currently no consensus on how to deal with *trans*-pQTLs, which are more prone to pleiotropy and in some cases are known to affect many proteins (examples include the *ABO* and *CFH* locus [82•]). *Cis*-only MR has been performed in some studies. However, incorporating bona fide *trans*-pQTLs could improve power. Tiered systems have been proposed to differentiate pQTLs according to their degree of pleiotropy and consistency between studies, and this can help to identify pQTLs more likely to meet the assumptions underlying MR [87]. Third, few studies have systematically performed the sensitivity analyses required to be able to discern between causality, reverse causality, and confounding by linkage disequilibrium (LD), i.e. the non-random inheritance of close-by single-nucleotide polymorphisms. The later can be assessed by colocalisation techniques to investigate if the protein and the outcome share the same causal variant. Finally, as large GWAS are becoming available for multiple complex traits, causal inference will be increasingly performed across a range of phenotypes, raising the question of candidate specificity. Several complex diseases share common pathogenic mechanisms, as proposed by the common soil hypothesis, postulating that the strong association between T2D and cardiovascular disease could be driven by genetic and environmental antecedents that are shared between diseases [89]. Phenome-wide MR studies will have the potential to discern between causal proteins specific for T2D and those linked to general pathogenic mechanisms involved in several diseases.

**Table 2:** Protein causal candidates for T2D, identified in high-throughput proteomic studies

Protein target	UniProt ID	# snps	Causal effect estimate (beta coefficient per SD increase in protein levels) (95% confidence interval)	Proteomic platform	Reference, year of publication
CFH	P08603	4	0.02 (0.005, 0.034)	SOMAscan	Preprint, [88], 2019
CFI	P05156	4	− 0.02 (− 0.03, − 0.003)	SOMAscan	Preprint, [88], 2019
SHBG	P04278	5	− 0.05 (− 0.07, −0.02)	SOMAscan	Preprint, [88], 2019
WFIKKN2	Q8TEU8	12	− 0.01 (− 0.02, − 0.004)	SOMAscan	Preprint, [88], 2019
COMT	P21964	1	− 0.11 (− 0.17, − 0.06)	Olink METABOLISM	Preprint, [90], 2020
ENTPD5	O75356	2	− 0.09 (−0.13, − 0.04)	Olink METABOLISM	Preprint, [90], 2020
LRIG1	Q96JA1	1	0.06 (0.04,0.08)	Olink METABOLISM	Preprint, [90], 2020
QDPR	P09417	2	− 0.10 (− 0.16, − 0.05)	Olink METABOLISM	Preprint, [90], 2020
TYRO3	Q06418	1	0.08 (0.05, 0.11)	Olink METABOLISM	Preprint, [90], 2020
CHI3L1	P36222	2	0.06 (0.02, 0.09)	Olink CVD-III	Preprint, [90], 2020
TNFRSF11A	Q9Y6Q6	2	− 0.06 (− 0.09, − 0.03)	Olink CVD-II	Preprint, [90], 2020
A4GALT	Q9NPC4	4	− 0.08 (− 0.12, − 0.03)	SOMAscan	Preprint, [59•], 2020
AMY2B	P19961	2	0.12 (0.05, 0.19)	SOMAscan	[59•], 2020
CCDC126	Q96EE4	6	0.08 (0.03, 0.12)	SOMAscan	[59•], 2020
COLEC11	Q9BWP8	16	− 0.02 (− 0.03, − 0.005)	SOMAscan	[59•], 2020
FAM177A1	Q8N128	6	− 0.03 (− 0.06, − 0.01)	SOMAscan	[59•], 2020
GDF15	Q99988	12	0.03 (0.014, 0.05)	SOMAscan	[59•], 2020
HIBCH	Q6NVY1	12	− 0.03 (− 0.05, − 0.01)	SOMAscan	[59•], 2020
KNG1	P01042	12	0.04 (0.02, 0.06)	SOMAscan	[59•], 2020
MLN	P12872	15	0.03 (0.01, 0.05)	SOMAscan	[59•], 2020
MMP12	P39900	14	− 0.03 (− 0.05, − 0.02)	SOMAscan	[59•], 2020
PLXNB2	O15031	9	− 0.06 (− 0.1, − 0.02)	SOMAscan	[59•], 2020
SEMA3G	Q9NS98	4	− 0.06 (− 0.1, − 0.02)	SOMAscan	[59•], 2020
SEMA4D	Q92854	13	− 0.02 (− 0.03, − 0.008)	SOMAscan	[59•], 2020
TNFSF12	O43508	6	− 0.02 (− 0.04, − 0.009)	SOMAscan	[59•], 2020
WFIKKN2	Q8TEU8	12	− 0.03 (− 0.04, − 0.01)	SOMAscan	[59•], 2020
PAPPA	Q13219	2	− 0.27 (− 0.42, − 0.11)	Olink CVD-I	Preprint, [84], 2020
RAGE	Q15109	2	− 0.17 (− 0.27, − 0.08)	Olink CVD-I	Preprint, [84], 2020

Polygenic scores based on disease variants have been alternatively used to identify disease-mediating candidate proteins. Ritchie et al. [88] evaluated the influence of genetic predisposition to complex diseases on the plasma proteome, identifying 48 proteins whose levels were modulated by polygenic scores for coronary artery disease, chronic kidney disease, and T2D. A large proportion of pQTLs (Emilsson V. et al. [83] report 60% of their discovery set) overlap with known disease-associated loci identified through GWAS, suggesting a common causal variant. However, in most cases where pQTLs overlap with variants composing disease GRSs (or with variants in high LD), associations were largely driven by polygenic effects rather than by these overlapping single loci [88].

## Conclusions

High-throughput proteomic technologies now provide the opportunity for large-scale hypothesis-free discovery of T2D plasma protein biomarkers and signatures, with specific technical and analytical challenges depending on the method used. Evidence for the predictive utility of novel protein biomarkers over and above established risk models is sparse, but larger prospective studies with improved analytical approaches are underway and anticipated to enable development of tailored risk assessment strategies for currently underdiagnosed subgroups. Integration of genomics and proteomics has the potential to provide technical validation, improve our understanding of the biological mechanisms linking genetic susceptibility to T2D, and prioritise causal pathways for intervention. Large population-based protein GWAS and validation of protein signals across diverse ancestries and proteomic platforms will be required to capitalise on the promise of early proof of concept studies and the potential of proteomics to contribute to the identification of novel and validation of existing therapeutic targets for T2D.
